# Quasi-freestanding graphene on Ni(111) by Cs intercalation

**DOI:** 10.1038/srep26753

**Published:** 2016-05-26

**Authors:** M. Alattas, U. Schwingenschlögl

**Affiliations:** 1 King Abdullah University of Science and Technology (KAUST), Physical Science and Engineering Division (PSE), Thuwal 23955-6900, Saudi Arabia

## Abstract

A possible approach to achieve quasi-freestanding graphene on a substrate for technological purpose is the intercalation of alkali metal atoms. Cs intercalation between graphene and Ni(111) therefore is investigated using density functional theory, incorporating van der Waals corrections. It is known that direct contact between graphene and Ni(111) perturbs the Dirac states. We find that Cs intercalation restores the linear dispersion characteristic of Dirac fermions, which agrees with experiments, but the Dirac cone is shifted to lower energy, i.e., the graphene sheet is n-doped. Cs intercalation therefore decouples the graphene sheet from the substrate except for a charge transfer. On the other hand, the spin polarization of Ni(111) does not extend through the intercalated atoms to the graphene sheet, for which we find virtually spin-degeneracy.

Graphene is gaining attention for its unique two-dimensional structure and special electronic properties, which are interesting for many applications^1^. A popular method to prepare graphene in high quality and large scale is chemical vapor deposition^2^, which has been successfully used for metallic substrates, such as Cu^3^, Au^4^, Co^5^, Ni^6^, Pd^7^, and Ir^8^. Ni(111) and Co(0001), in particular, are considered to be good substrates due to their small lattice mismatch of only 1.3%^9^ and 1.9%^10^, respectively. However, some metallic substrates, such as Ni(111)^11^ interact strongly with graphene and distort the Dirac cone^12^, whereas for Pt(111)^13^, for example, the interaction is weaker. The small distance (2.1 Å) realized between graphene and Ni(111) results in significant hybridization of the Ni 3d and C 2p orbitals, which explains the strong modification of the band structure^14^,^1^5. It has been shown experimentally^16^ and theoretically^17^ that C magnetic moments are induced.

Intercalation of noble metal atoms can restore the original band structure to give rise to quasi-freestanding graphene^18^. Ag intercalation into the graphene/Ni(111) system has been studied by density functional theory and it has been found that the Ag atoms decouple graphene from the substrate electronically^19^. The band gap decreases when the Ag concentration grows, as the interaction with the substrate is reduced. Ni then has no other role than to support the noble metal atoms. For Au intercalation the concentration also has a noticeable impact on the shape of the graphene *π* bands^20^. A low Au concentration of ~0.33 monolayers largely recovers the Dirac cone but leaves a band gap of 0.2 eV and a sizable shift of the Dirac point by 0.48 eV below the Fermi level. Increasing the Au coverage, on the other hand, almost removes the band gap and shifts the Dirac cone back to the Fermi level. Metal intercalation is not restricted to noble metals. In particular, alkali metals have been studied experimentally^11^ and theoretically^21^. They also weaken the interaction between graphene and Ni(111) by enlarging the interlayer distance^22^,^2^3.

In the following, we consider the structural, electronic, and magnetic properties of graphene on Ni(111), using density functional theory, and analyze the changes when intercalating the system with different concentrations of Cs atoms. By the large atomic radius of Cs a very efficient decoupling of graphene from the substrate can be expected. Cs is an alkali metal with one electron in its outer shell and thus strongly favors a Cs^+1^ state, so that it does not participate in chemical bonding but acts purely as spacer.

## Results

**Figure 1:**
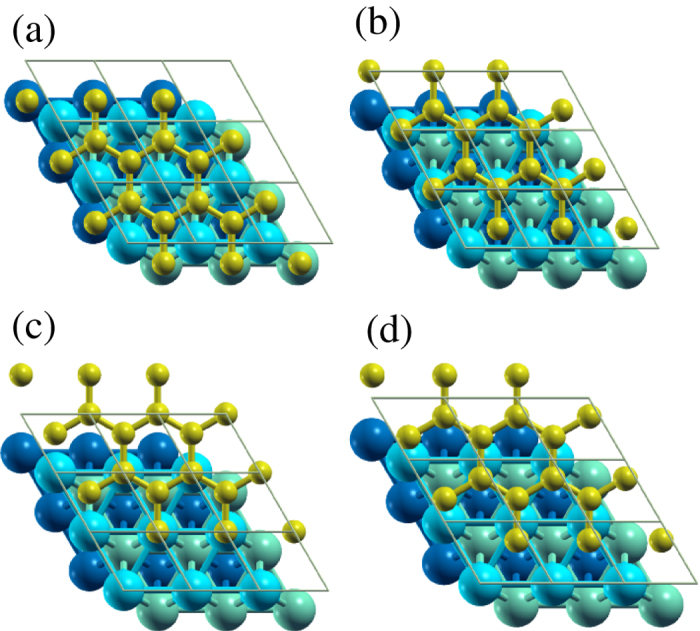
Top view of the structural configurations considered for graphene on Ni(111), where the C atoms (yellow) occupy (**a**) hollow, (**b**) fcc, (**c**) hcp, and (**d**) bridge sites on the Ni(111) surface.

The 1 × 1 unit cell of the graphene on Ni(111) consists of 4 Ni layers and 1 C layer on top. To determine the favorable lateral shift between the two materials, we study four structural configurations: (i) Hollow, where each atom of the topmost Ni layer is in the center of a C honeycomb. (ii) Face centered cubic (fcc), where three of the C honeycomb atoms are located on top of atoms of the topmost Ni layer and the center of the C honeycomb is located on top of an atom of the second Ni layer. (iii) Hexagonal close packed (hcp), which is similar to the fcc case but with the center of the C honeycomb located on top of an atom of the third Ni layer. (iv) Bridge, where the center of the C honeycomb is located between two atoms of the topmost Ni layer. Figure 1 illustrates the four cases and Table 1 summarizes key findings. In agreement with previous studies^24^, the fcc configuration turns out to have the lowest formation energy


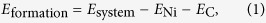


where *E*_Ni_ is the total energy per atom in bulk Ni and *E*_C_ is the total energy per C atom in graphene. The fact that the bridge configuration is only 10 meV higher in energy than the fcc configuration implies that it is accessible as well. Even the hcp configuration might be accessible with low probability. Indeed, these configurations experimentally can coexist^25^,^2^6. The hollow configuration, on the other hand, is rather unlikely to be formed.

**Figure 2:**
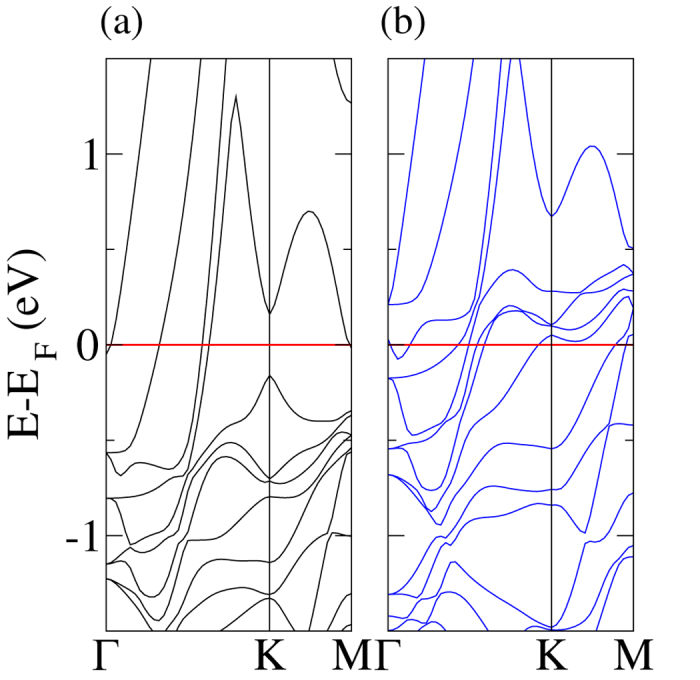
Graphene on Ni(111): Band structure of the (**a**) spin up and (**b**) spin down channels.

The C-C bond length in the fcc configuration is 1.44 Å, whereas it is 1.42 Å in pristine graphene. This small tensile strain corresponds to the mentioned small lattice mismatch. The obtained perpendicular distance between graphene and the Ni(111) surface, *d*_C/Ni_, see Table 1, is in very close agreement with previous experimental^27^ and theoretical^28^ findings. For the fcc configuration, Fig. 2 shows the band structure for the spin up and down channels. The Dirac cone is strongly perturbed with a 0.3 eV splitting at the K point, reflecting a strong hybridization between the C 2p and Ni 3d states as a consequence of the small *d*_C/Ni_. We obtain finite though small magnetic moments of −0.02 and 0.03 *μ*_*B*_ on the two C atoms due to Ni-C hybridization, in agreement with the results reported in ref. 29, and charge transfers of 0.03 and −0.01 electrons from the Ni 3d to the C 2p states.

**Figure 3:**
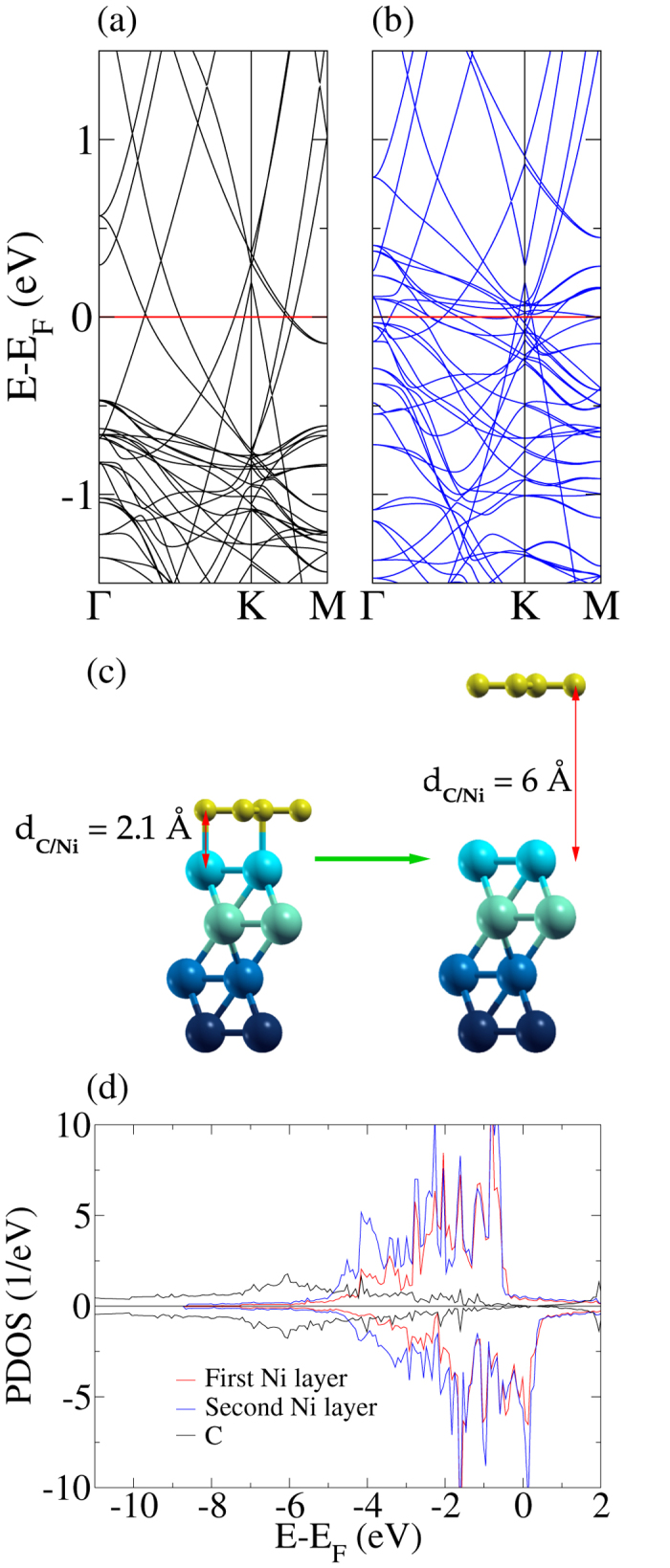
Band structure of the (**a**) spin up and (**b**) spin down channels for *d*_C/Ni_ = 6 Å. (**c**) Atomic structure with real and artificially exaggerated distance between the graphene sheet and Ni substrate. (**d**) Projected densities of states.

We next consider a 2 × 2 supercell with graphene artificially placed in a distance of 6 Å from the Ni(111) substrate, see Fig. 3(c). As expected, this distance yields a weak interaction between the Ni 3d and C 2p states, compare the almost vanishing Ni-C hybridization, leading to a restoration of the Dirac cone with a minor splitting. In fact, hybridization effects start vanishing in distances larger than 3 Å. Figure 3(a,b) shows the band structures of the spin up and down channels. We obtain a shift of the Dirac point by 0.2 eV above the Fermi level, reflecting p-doped graphene. The Dirac cone appears in the two spin channels at the same energy, implying that there is no C magnetic moment. The Ni bands, on the other hand, show a rigid shift due to spin polarization. Projected densities of states in Fig. 3(c) confirm that the magnetic moments on the C atoms are zero and that the total magnetic moment of 10.8 *μ*_*B*_ comes from the Ni atoms, where atoms in the first layer show magnetic moments of 0.67 *μ*_*B*_ and atoms in the other layers show 0.68 *μ*_*B*_.

**Figure 4:**
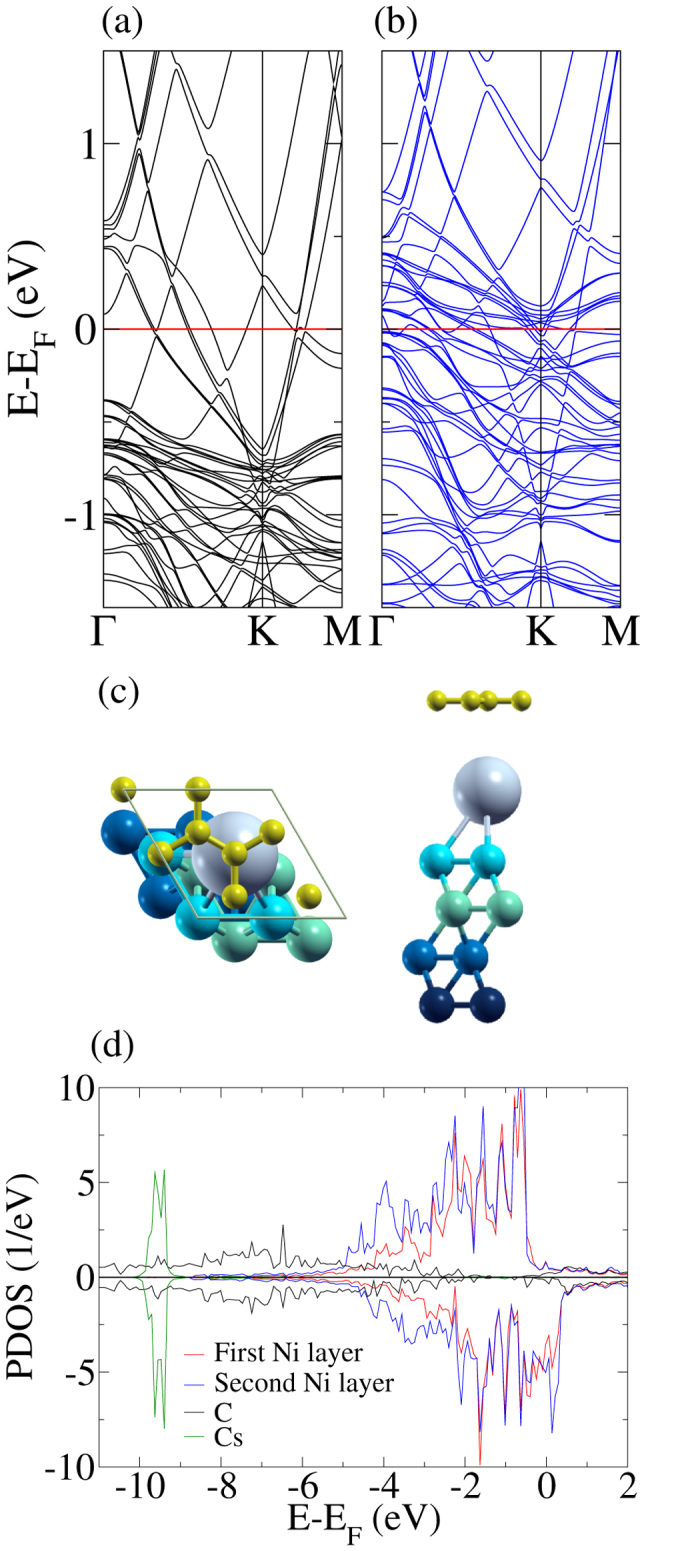
Band structure of the (**a**) spin up and (**b**) spin down channels for the graphene/Cs/Ni(111) system (2 × 2 supercell). (**c**) Atomic structure with intercalated Cs (gray). (**d**) Projected densities of states.

**Figure 5:**
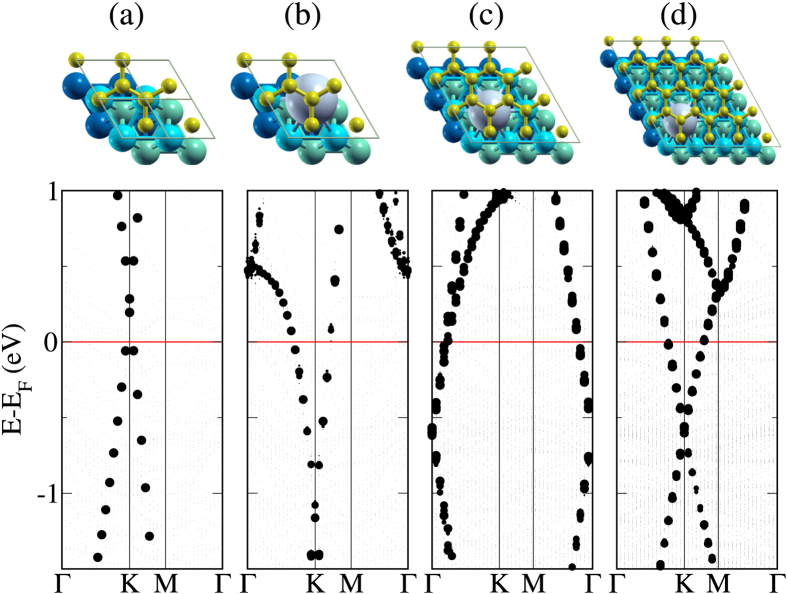
Atomic structures and weighted bands for the (**a,b**) 2 × 2 supercell without/with intercalation, (**c**) 3 × 3 supercell, and (**d**) 4 × 4 supercell. The size of the dots represents the sum of all C *p*_*z*_ contributions.

We find that intercalation of Cs enlarges the distance between the graphene sheet and the Ni(111) substrate to 6.0 Å post relaxation (3.1 Å from graphene to Cs and 2.9 Å from Cs to Ni). The in-plane Cs-Cs distance is 5.0 Å. We note that the graphene sheet shows no significant structural modification, see Fig. 4(c), in particular hardly any buckling. According to Fig. 4(a,b), the electronic structure, on the other hand, is altered substantially by the Cs intercalation. In particular, a multitude of bands appears from −1.5 to 0.5 eV. To understand the origin of these bands we use a weighted band analysis. Figure 5(a) shows that the cone-like bands originate from the C 2p states, while the other bands are due to the Ni 3d states. The Dirac cone is restored with a negligible splitting. It appears about 1.1 eV below the Fermi level, representing prominently n-doped graphene. The C atoms carry no magnetic moment, see also Fig. 4(d), implying that Cs does not transfer spin polarization from Ni to graphene. In the Ni(111) substrate the magnetic moments decrease towards Cs from a value of 0.68 *μ*_*B*_, which is close to the bulk Ni value of 0.67 *μ*_*B*_, to values of 0.49 *μ*_*B*_ for the atom directly below Cs and 0.59 *μ*_*B*_ for the other atoms in the same layer.

We next consider larger supercells, see Fig. 5 on top, to study the effect of the Cs concentration. The weighted band structures obtained for 2 × 2 (without and with intercalation), 3 × 3, and 4 × 4 supercells in Fig. 5 demonstrate a shift of the Dirac point upwards to the Fermi level when the Cs concentration is reduced. The higher the Cs concentration the stronger is the n-doping of graphene. The Dirac point is located 1.12 eV, 0.61 eV, and 0.59 eV below the Fermi level, respectively, for the 2 × 2, 3 × 3, and 4 × 4 supercell, which corresponds to a charge transfer of 0.045, 0.012, and 0.011 electrons in the case of pristine graphene.

**Figure 6:**
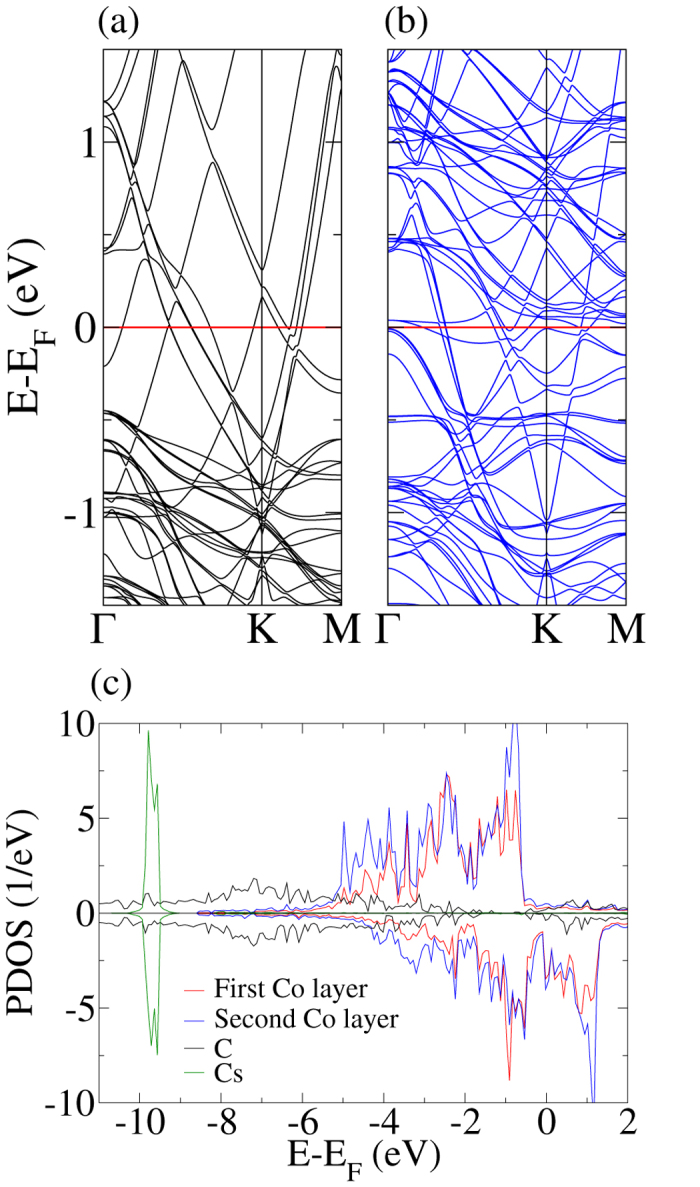
Band structure of the (**a**) spin up and (**b**) spin down channels for the graphene/Cs/Co(0001) system (2 × 2 supercell). (**c**) Projected densities of states.

As another ferromagnetic substrate we have also considered Co(0001) to check whether our results for Ni(111) are of general validity. The band structure of the 2 × 2 supercell, see Fig. 6(a,b), is found to show close similarity to the case of the Ni(111) substrate, only the Dirac point is shifted slightly more (1.17 eV) below the Fermi level. The projected densities of states in Fig. 6(c), on the other hand, indicate that Co is subject to a stronger exchange splitting as compared to Ni.

## Discussion

We have investigated the effect of Cs intercalation on the structural, electronic, and magnetic properties of graphene on Ni(111) and Co(0001). An fcc configuration is found to be energetically favorable in agreement with previous experimental and theoretical results, and therefore is chosen for studying the intercalation. Different supercell sizes have been considered to examine the effect of the Cs concentration. In agreement with experimental results, the Cs intercalation restores the Dirac cone, since it decouples graphene from the substrate, resulting in n-doped quasi-freestanding graphene. Both for the Ni(111) and Co(0001) substrates the graphene sheet exhibits no trace of spin polarization.

## Methods

Density functional theory is employed using the Vienna Ab-initio Simulation Package^30^ and the generalized gradient approximation (Perdew-Burke-Ernzerhof parametrization^31^) along with ultrasoft pseudopotentials. London forces are considered in all calculations^32^ and the plane wave cutoff energy is set to 500 eV. Supercells are created using in the xy-plane the lattice parameter of Ni (2.49 Å) and adding a vacuum slab of about 15 Å thickness in the z-direction. For a 1 × 1 unit cell of graphene on Ni(111) a 32 × 32 × 1 k-mesh (Monkhorst-Pack scheme^33^) is used for the Brillouin zone integration (16 × 16 × 1 for the structure relaxation). We built a 2 × 2 supercell containing 24 atoms (8 C and 16 Ni) and introduce one Cs atom, giving a total number of 25 atoms. Also 3 × 3 and 4 × 4 supercells are constructed to examine the effect of the Cs concentration. These supercells are relaxed using 4 × 4 × 1 and 2 × 2 × 1 k-meshes, respectively, and 8 × 8 × 1 and 4 × 4 × 1 k-meshes are employed for the self-consistency calculation.

## Additional Information

**How to cite this article**: Alattas, M. and Schwingenschlögl, U. Quasi-freestanding graphene on Ni(111) by Cs intercalation. *Sci. Rep.* **6**, 26753; doi: 10.1038/srep26753 (2016).

## Figures and Tables

**Table 1:** Comparison of different configurations of graphene on Ni(111).

	hollow	fcc	hcp	bridge
*d*_C/Ni_ (Å)	3.3	2.1	3.0	2.2
*E*_formation_ (eV)	−4.01	−4.19	−4.11	−4.18
